# Maintaining mask stockpiles in the COVID-19 pandemic: Taiwan as a learning model

**DOI:** 10.1017/ice.2020.226

**Published:** 2020-05-11

**Authors:** Cho-Hung Chiang, Cho-Hsien Chiang, Cho-Han Chiang

**Affiliations:** 1 Department of Medicine, College of Medicine, Fu-Jen Catholic University, New Taipei City, Taiwan; 2 School of Medicine, Chung Shan Medical University, Taichung City, Taiwan; 3 Department of Medicine, College of Medicine, National Taiwan University, Taipei, Taiwan

*To the Editor*—The US Centers for Disease Control and Prevention (CDC) recently recommended the use of face masks in public settings to help slow the spread of the SARS-Cov-2 virus.^1^ Currently, a mask shortage continues in the United States and around the globe. In contrast, Taiwan has had a surplus of masks since February and exported masks in April to other countries to help fight this pandemic. In this letter, we discuss how Taiwan ensured adequate stockpiles of masks for both healthcare workers and the general population.

**Fig. 1.**
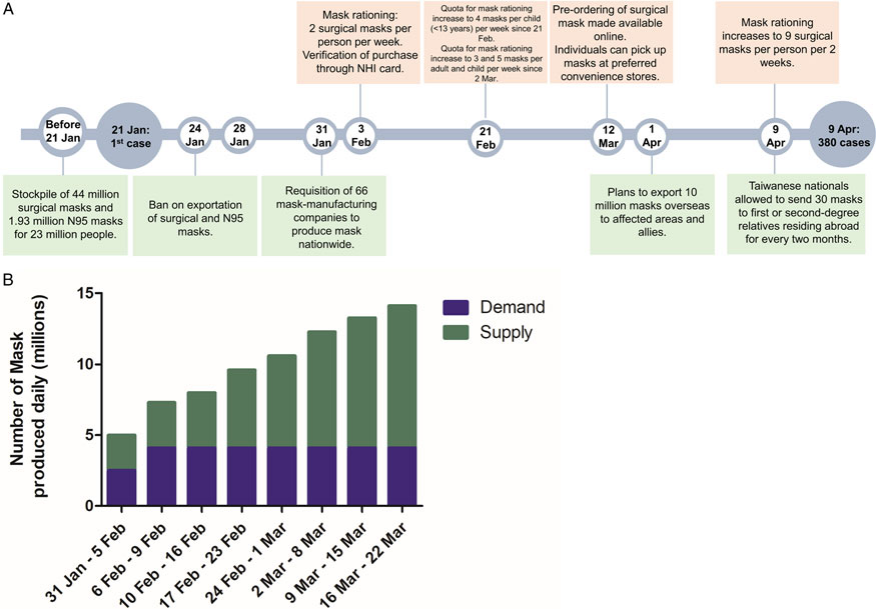
(A) Timeline summarizing the policies implemented by the Taiwanese government to maintain adequate stockpiles of mask. (B) Demand and supply of mask by weeks.

Taiwan registered its first case of COVID-19 on January 21, 2020.^2^ As of April 17, 2020, Taiwan has recorded just 395 cases of COVID-19. In response to the emerging threat of COVID-19, the Taiwanese government produced a stockpile of 44 million surgical and 1.93 million N95 masks before the first case was reported (Fig. 1A).^3^ Subsequently, the government implemented bans on exportation and requisition of mask-manufacturing companies to ensure adequate local reserves of masks. The government also introduced price fixing of masks to prevent bidding wars and to ensure universal access of masks to all citizens. Rationing of surgical masks was implemented to prevent panic buying of masks, which would drain local supplies. The mask rationing system was delicately adjusted according to the production of masks and the amount required by healthcare workers, while ensuring that the entire population had adequate access (Fig. 1A).

The initial rationing system limited every person to 2 surgical masks per week. The purchase was tracked using the individual’s national health insurance card—health insurance that covers 99% of residents. With the resumption of school classes on February 21, the purchase limit for surgical masks was increased to 4 masks per week for children (Fig. 1A).^4^ With the increased production of masks, the quota increased to 5 masks for children and 3 masks for adults per week. By the end of February, Taiwan was producing 5 million masks daily and had a substantial surplus of masks (Fig. 1B).^4^ As of April 9, 2020, the purchase quota was increased to 9 surgical masks per person per 2 weeks. The ban on the export of masks was lifted, and Taiwanese nationals can now send masks to relatives residing abroad. To help the rest of the world to battle the pandemic, Taiwan also exported 10 million masks to the United States, Europe, and its allies in April 2020.^4^

Since the outbreak, many Asian countries have recommended or mandated the use of surgical masks in the healthy general population.^5^ Previously, the US CDC recommended that masks should only be worn by healthcare workers, caregivers, or symptomatic individuals. Part of this rationale was that universal masking in the general population may limit the amount of masks available to healthcare settings. However, Taiwan’s example illustrates that this supply problem may be overcome through a combination of policies such as increased mask production and rationing, and ensuring that the demand and supply of masks remain in balance (Fig. 1B).

Although the use of surgical masks in the healthy population has not been directly proven to reduce the spread of the coronavirus, it may provide source control and protect against asymptomatic transmission.^5^ Recent mechanistic studies have suggested that surgical masks may reduce the transmission of the coronavirus if worn by patients.^6^ Furthermore, a recent meta-analysis showed that masking practices may produce marginal reduction in transmission of respiratory virus in the community.^7^ At a population level, even marginal reduction in transmission may substantially slow the spread of the SARS-CoV-2 virus. Economically, mask interventions have been shown to be cost saving in a pandemic setting.^8^ Taiwan created a sufficient stockpile of masks for both the healthcare settings and the general population, and its example could serve as a model for other countries to learn from as we continue our fight against the COVID-19 pandemic.
